# Lesbian, Gay, and Bisexual Students Experiencing Homelessness and Substance Use in the School Context: A Statewide Study

**DOI:** 10.1111/josh.13246

**Published:** 2022-08-15

**Authors:** Hadass Moore, Kris De Pedro

**Affiliations:** ^1^ The Paul Baerwald School of Social Work and Social Welfare Hebrew University of Jerusalem Jerusalem Israel; ^2^ Attallah College of Educational Studies Chapman University Orange CA

**Keywords:** student homelessness, substance use, school health, homeless youth, alcohol, tobacco, other drugs, LGB students

## Abstract

**PURPOSE:**

This study explored differences between lesbian, gay, and bisexual (LGB)‐housed and homeless students regarding substance use patterns on and off school grounds and the unique contribution of homelessness to substance use in school.

**METHODS:**

Data were from the 2013‐2015 California Healthy Kids Survey, a statewide survey of school protective factors and risk behaviors. A representative sample of 9th‐ and 11th‐grade students (N = 20,337) was used. Comparisons between housed (n = 19,456) and homeless (doubled up: n = 715; acute homeless: n = 166) LGB students were conducted. We used chi‐square tests to compare rates of lifetime, past‐30‐day, and in‐school substance use and conducted multivariate logistic regression models for each substance use variables.

**RESULTS:**

Chi‐square test results indicated significant differences in rates of substance use among students experiencing homelessness (both categories) and housed LGB students. Lesbian, gay, and bisexual students experiencing homelessness were more likely to report substance use off and on school grounds. Results from logistic regression analyses indicated that LGB students who experience homelessness were significantly more likely to report recent and in‐school substance use. For example, students experiencing acute homelessness were about 3 times as likely to report heavy episodic drinking (adjusted odds ratio [AOR] = 3.13; 95% confidence interval [CI] = 2.13, 4.26) and more than 5 times as likely to smoke marijuana in school (AOR = 5.38; 95% CI = 3.46, 8.36), compared to housed LGB students.

**CONCLUSIONS:**

LGB students who experience homelessness are at higher risk than housed LGB students of substance use on and off school grounds. Findings highlight the need to provide support in the school context for this subpopulation.

Youth homelessness is a significant public health challenge in the United States. Previous studies show that multiple adverse behavioral, social, and health problems are associated with youth homelessness.^1^, ^2^ Many of these youth attend schools, at least sporadically; federal education data shows that more than 1.5 million homeless students are presently enrolled in schools across the country.^3^ Still, the school context and its role for homeless youth is frequently overlooked in the research literature.^4^, ^5^ Additionally, evidence shows that lesbian, gay, and bisexual (LGB) youth are overrepresented among the homeless youth population compared with the general youth population.^6^, ^7^ Despite the growing literature on LGB youth homelessness in the past decade,^6^, ^7^, ^8^ LGB students who experience homelessness represent a distinct subpopulation of the homeless youth population.^9^, ^10^ The intersection among youth homelessness, sexual orientation, and the school context is missing from the research literature, specifically regarding risk behaviors. Moreover, the use of school‐based data from LGB homeless youth in relation to adverse outcomes is limited.^11^ The literature is particularly limited in terms of comparing housed students and students experiencing homelessness in the LGB student population on issues of substance use on school grounds. The current study aimed to address this gap by examining differences between housed LGB students and LGB students experiencing homelessness, on issues of substance use on and off school grounds using a large‐scale, statewide sample of LGB high school students in California.

Research from the past 3 decades consistently documented high rates of substance use for both homeless youth^1^, ^2^, ^12^, ^13^ and LGB youth.^14^, ^15^, ^16^ The fact that LGB youth have significantly higher substance use rates than their non‐LGB peers has been well supported in the literature and often attributed to sexual minority stress.^16^ According to the sexual minority stress prism, LGB youth experience unique stressors due to their stigmatized sexual identities.^16^, ^17^ While considering the intersection of youth homelessness, housing instability, and sexual orientation, homelessness may be considered a minority stress factor.^6^ For LGB youth, homelessness is often related to their sexual minority status; family conflict over sexual orientation and family rejection leads some LGB youth to being forced out of their homes or run away.^9^, ^18^ Additionally, studies show that LGB youth are more subjected to childhood abuse compared to heterosexual persons, which also may lead to homelessness as a coping strategy.^18^, ^19^ Another reason for homelessness among LGB youth is aging out of the foster care system, where they often experience harassment and violence.^18^, ^19^ Hence, the unique condition of LGB homeless youth deserves special consideration in prevention and intervention efforts. Lesbian, gay, and bisexual homeless youth as a subgroup of the homeless youth population face greater risk of mental health problems and substance use compared to their heterosexual peers.^9^, ^20^ They experience higher rates of family rejection, discrimination, victimization, and trauma, which can contribute to low self‐esteem and the development of mental health problems.^9^, ^20^ In an effort to cope, LGB homeless youth may engage in substance use.^16^ A study that compared housed LGB youth and LGB youth who experience homelessness found that substance use was more frequent and first occurred at an earlier age in homeless LGB youth.^6^

Regarding the school context, there is a scarcity in the literature when it comes to the unique population of students experiencing homelessness and LGB homeless students in particular.^5^ Research shows that homeless students are underidentified and underserved.^5^, ^21^ This is often related to the challenge of identification and lack of awareness of the definition of homelessness established by the education subtitle of the McKinney‐Vento Homeless Assistance Act (MVA),^22^ which provides a broader, more nuanced understanding of homelessness and housing instability. Specifically, it states that a student who lacks a fixed, regular, and adequate nighttime residence is considered homeless. The inclusion of multiple subgroups in the homeless youth population under this definition provides an opportunity to support and intervene with homeless LGB students through the school context before they disengage from all social institutions. The school is a valuable context to explore for these students, especially considering substance use that occurs on school grounds. The little existing research has identified that homeless students report higher rates of substance use on and off school grounds compared with their housed peers.^23^, ^24^ Additionally, research shows that LGB students use substances at higher rates than their heterosexual peers.^16^ Schools are central locations in the lives of LGB students who experience homelessness as they may be the last social institution they engage with prior to disengaging all social institutions.^5^, ^9^ Studies show that high‐school students are exposed to, buy, sell, as well as use substances in school.^25^, ^26^ A recent national survey found that among students who reported personally seeing substance use, their own schools were the most frequent place reported to witnessing substance use.^26^ This means that schools can be a risk factor for LGB students experiencing homelessness as a place of exposure to substances as well as initiation and use of substances.^24^, ^25^, ^26^ Moreover, studies also document the potential protective role that schools, and school programs can have on youths' substance use.^24^ Therefore, there is a need to explore the intersection of homelessness, sexual orientation, substance use, and schools, both due to this unique population and in relation to school as a context for these youth.

In light of policy amendments under the Every Student Succeeds Act (ESSA)^27^ regarding issues of accountability, socioemotional learning standards, and homelessness, an urgent need exists to focus on LGB homeless students as an at‐risk population and schools as sites of investigation for advancing educational opportunities for LGB homeless students. This study utilized a statewide sample, and as such, serves as the initial step in understanding the unique needs of LGB homeless students from an epidemiological standpoint while considering implications for advancing health and equal access to education for LGB homeless students.

## Current Study

The present study had several aims. First, it sought to examine differences between homeless and housed LGB students in the patterns of substance use (lifetime and recent use) while considering subgroups of LGB homeless students as established in the MVA definition. Second, it examined differences between homeless and housed LGB students in substance use issues on school grounds while considering subgroups of LGB homeless students. Third, it explored the relationship between homelessness and substance use among LGB students, controlling for student demographics. Fourth, this study explored if and to what degree homelessness adds to the risk of substance use among LGB students. Given the gap in the research, this study drew from a statewide sample of LGB students in California to address the study's aims. Findings from this study can be used to inform substance use prevention programs in schools and provide meaningful information for educators, researchers, and policy makers who are interested in advancing health, well‐being, and equal opportunity of education for all.

## METHODS

### Participants and Procedures

The current analysis drew from 2013‐2015 California Healthy Kids Survey (CHKS). The CHKS is the largest statewide survey of school protective factors, safety, and risk behaviors in the United States. The CHKS is administered biennially to students in 5th, 7th, 9th, and 11th grades from all school districts throughout California.^28^ The CHKS includes reliable scales on school connectedness and substance use items and gathers information on student demographics, including sexual orientation, race and ethnicity, gender, and grade level. In addition, data from the CHKS are student reported.^28^ Measures in the CHKS are similar to items used in the Centers for Disease Control and Prevention's Youth Risk Behavior Surveillance System for adolescent risk behaviors. School districts obtained appropriate consent and assent from parents and student participants, and the study was reviewed by the California Department of Education and West Ed. This study involved secondary analyses of existing publicly available data with no identifiers provided by the California Department of Education and WestEd. Therefore, this study did not require oversight or review by the Institutional Review Board.

Data analyses focused on a subsample of LGB youth (N = 20,337). The CHKS module for fifth grade does not include a question about LGB identity. In addition, the CHKS module administered in fifth and seventh grades does not include a question assessing homelessness. Hence, fifth‐ and seventh‐grade participants were excluded from the study.

### Dependent Variable

#### Substance use

To address the aims of this study, this study included items measuring lifetime, past‐30‐day, and in‐school substance use rates. All substance use items were treated as dichotomous variables, with students reporting whether they had used each item. Substances assessed included alcohol, tobacco, cigarettes, marijuana, inhalants, prescription painkillers (Vicodin, OxyContin, Percodan, Lortab, tranquilizers, Xanax, or Ativan), and others.

### Independent Variables

#### Demographic characteristics

The CHKS also asks students to report the following demographic characteristics: sex, race and ethnicity, sex assigned at birth, grade level, and parental educational attainment. The CHKS asks students, “Which of the following best describe you? (mark all that apply).” The item response categories included heterosexual (straight), gay or lesbian, or bisexual, transgender, not sure, and decline to respond. The study included participants who marked “gay or lesbian or bisexual.” The item assessing sex included 2 responses (male, female). Race and ethnicity were assessed utilizing white as the reference category with the following dummy variables (American Indian, Asian or Pacific Islander, black, multiracial, and Latino). In addition, students reported the highest level of education of the parent who completed the most schooling. The response categories were did not finish high school, graduated from high school, attended college but did not complete a 4‐year degree, and graduated from college. Grade level included 2 categories (9th grade, 11th grade).

#### Homelessness

The CHKS included an item asking students to report their living situation: “What best describes where you live? A home includes a house, apartment, trailer, or mobile home.” The responses included a home with 1 or more parent or guardian; other relative's home; a home with more than 1 family; a friend's home, foster home, group care, or waiting placement; hotel or motel; shelter, car campground, or other transitional or temporary housing; and other living arrangement. For this study and based on the MVA definition, students were classified into 2 categories of homelessness: (a) students who lived in a shelter, car, campground, other transitional or temporary housing, hotel, or motel (acute homelessness); and (b) students who lived in another relative's or friend's home (doubled up). Participants classified as housed included students who reported that they lived in a home with 1 or more parent or guardian, a home with more than 1 family, or another living arrangement.

### Analytic Plan

To address the study's objectives, bivariate and multivariate analyses were conducted using IBM SPSS version 25. Descriptive statistics were conducted to assess sample characteristics, including race and ethnicity, grade level, parent education level, sex, and homelessness. Next, we performed cross‐tabulations and chi‐square tests of association to compare rates of lifetime, past‐30‐day, and in‐school substance use among homeless and housed youth in the LGB population. We then conducted multivariate logistic regression models for each substance use variable. The regression models controlled for race and ethnicity, sex, and parent education level and assessed odds of lifetime, past‐30‐day, and in‐school substance use among homeless (doubled up and acute homeless) LGB youth compared to housed LGB students.

## RESULTS

Univariate statistical analyses indicated a racially diverse sample of LGB youth. As presented in Table 1, more than three‐quarters of the sample was non‐White. Asian and Pacific Islander students comprised 9.2% of the sample, whereas 4.4% and 11.9% of LGB students identified as black and multiracial, respectively. In addition, more than half of the overall sample was classified as Latino (51.0%). More than half of the doubled up and acute homeless students also identified as Latino (55.9% and 50.3%, respectively).

**Table 1:** Overall Sample Characteristics

	Overall (N = 20,337)	Doubled Up (n = 715)	Acute Homeless (n = 166)	Housed (n = 19,456)
Variable	n (%)	%	%	%
Sex (n = 19,959)				
Male	29.9	24.4	45.6	30.0
Female	70.1	75.6	54.4	70.0
Grade (n = 20,337)				
9	49.5	48.8	53.6	49.4
11	50.5	51.2	46.4	50.6
Race and ethnicity (20,011)				
Asian or Pacific Islander	9.2	5.3	9.4	9.4
American Indian	0.9	2.1	1.3	0.9
Black	4.4	6.4	10.1	4.2
White	22.5	17.1	17.6	22.9
Multiracial	11.9	13.1	11.3	11.9
Latino	51.0	55.9	50.3	50.8
Parent education (17,629)				
Less than high school	19.7	28.4	48.1	19.0
High school graduate	20.9	24.7	16.3	20.7
Some college	18.9	21.7	13.2	18.9
College graduate	40.5	25.2	22.5	41.3

The sample was composed of 70.1% female students and 29.9% male students. The analyses indicated that 0.8% of the LGB sample (n = 166) reported acute homelessness and about 3.5% of LGB students reported being doubled up (n = 715).

Table 2 presents the rates of lifetime, past‐30‐day and in‐school substance use among housed, doubled up, and students who experience acute homelessness youth. The overall sample indicated higher substance use rates compared to recent national data on adolescent substance use.^26^ For instance, 25.4% of participants reported past‐30‐day marijuana use compared to 6.9% in the national data.^26^ Additionally, many of the participants reported substance use on school grounds, for example. About two thirds (66.5%) of participants reported using other illegal drugs in school in the past 30 days.

**Table 2:** Rates of Lifetime, Past‐30‐Day and In‐School Substance Use Among Housed, Doubled Up, and Acute Homeless Youth

	Overall (N = 20,337)	Doubled Up (n = 715)	Acute Homeless (n = 166)	Housed (n = 19,958)	Pearson Chi‐Square (df)
Substance	%	%	%	%	
Lifetime					
Cigarettes* (n = 20,173)	26.6	39.2	42.1	26.0	81.2 (2)
Tobacco* (n = 20,237)	6.0	10.5	23.5	5.7	115.6 (2)
Alcohol* (n = 20,251)	56.7	66.3	69.1	56.2	39.7 (2)
Marijuana* (n = 20,234)	45.3	58.8	57.6	44.6	65.5 (2)
Inhalants* (n = 20,281)	14.1	20.6	29.9	13.7	61 (2)
Cocaine or methamphetamine* (n = 20,292)	7.1	12.9	27.7	6.7	148.2 (2)
Ecstasy* (n = 20,242)	11.1	16.8	35.5	10.7	125.9 (2)
Prescription pain medication* (n = 20,267)	24.3	35.3	38.0	23.8	65.9 (2)
Ritalin or Adderall* (n = 20,181)	8.9	11.9	26.7	8.6	74.6 (2)
Cold medicine* (n = 20,230)	45.4	55.2	49.1	45.0	29.8 (2)
Other drug* (n = 20,235)	17.6	26.2	37.3	17.1	84.6 (2)
Past 30 days					
Cigarettes* (n = 20,201)	12.2	24.9	34.5	11.7	111.1 (2)
Tobacco* (n = 20,234)	2.7	3.8	22.0	2.5	237.4 (2)
Alcohol (1 drink)* (n = 20,204)	32.8	40.1	50.9	32.3	43.6 (2)
Heavy episodic drinking* (n = 20,235)	18.4	26.9	37.7	18.0	76.8 (2)
Marijuana* (n = 20,224)	25.4	38.1	47.6	26.0	97.5 (2)
Inhalants*3	5.5	8.0	24.2	5.1	130.4 (2)
Prescription pain medication*(n = 20,263)	9.2	15.5	29.7	8.8	119.7 (2)
Other drug* (n = 20,250)	8.2	14.4	26.7	7.8	114.6 (2)
In school					
Cigarettes* (n = 20,245)	3.7	7.0	20.1	3.4	150.5 (2)
Tobacco* (n = 20,214)	2.2	4.2	17.4	2.0	194.7 (2)
Alcohol* (n = 20,202)	9.3	14.6	30.1	9.1	108.7 (2)
Marijuana* (n = 20,245)	5.4	9.3	24.1	5.1	133.9 (2)
Other illegal drugs (n = 20,143)	66.5	67.1	67.1	66.5	.2 (2)

* *p* < .001.

### Bivariate Analyses

As seen in Table 2, chi‐square tests of association indicated significant differences in rates of substance use among homeless (both categories) and housed LGB students. About 42.1% of acute homeless LGB students and 39.2% of doubled‐up LGB students reported lifetime cigarette use, compared to 26.1% of housed LGB students. In addition, 27.7%% of acute homeless LGB students and 12.9% of doubled‐up LGB students reported lifetime cocaine or methamphetamine use, compared to 7.3% of housed LGB students. The results also indicated significantly higher rates of past‐30‐day prescription pain medication use among acute homeless LGB students (29.7%) and doubled‐up LGB students (15.5%), when compared to housed LGB students (8.9%). In addition, homeless LGB students use substances in school at higher rates than their housed peers. For example, 30.1% of acute homeless LGB students and 14.6% of LGB doubled‐up students reported drinking alcohol at school, compared to 9.1% of housed LGB students.

### Multivariate Logistic Regression Analyses

Results from the lifetime substance use multivariate regression models (Table 3) indicated that each category of homeless LGB students were more likely to use all substances in their lifetime when compared to housed LGB students, controlling for grade level, biological sex, and race and ethnicity. Acute homeless LGB students had the highest risk. For instance, acute homeless LGB students were almost 5 times as likely to report lifetime tobacco use (adjusted odds ratio [AOR] = 4.72; 95% confidence interval [CI] = 3.04, 7.31), about 6 times as likely to report lifetime cocaine and methamphetamine use (AOR = 6.25; 95% CI = 4.16, 9.39), and more than 2 and a half times as likely to report lifetime prescription pain medication use (AOR = 2.75; 95% CI = 1.89, 4.02) compared to their housed peers. In addition, doubled‐up LGB students were 30% more likely to report lifetime alcohol use (AOR = 1.29; 95% CI = 1.08, 1.55) and about 46% more likely to report lifetime ecstasy use (AOR = 1.46; 95% CI = 1.16, 1.85), when compared to housed LGB students.

**Table 3:** Adjusted Odds Ratios and CI From Logistic Regression Analyses Examining Past‐30‐Day and In‐School Substance Use Among Doubled‐Up and Acute Homeless Youth

Substance	Doubled Up (n = 715)	Acute Homeless (n = 166)	Valid Cases
Lifetime			
Cigarettes	1.71* (1.43, 2.04)	2.70* (1.84, 3.95)	16,765
Tobacco	1.69* (1.27, 2.26)	4.72* (3.04, 7.31)	16,820
Alcohol	1.29* (1.08, 1.55)	2.51* (1.64, 3.85)	16,833
Marijuana	1.53* (1.29, 1.83)	2.11* (1.43, 3.12)	16,816
Inhalants	1.36* (1.09, 1.68)	2.55* (1.69, 3.86)	16,856
Cocaine or methamphetamine	1.78* (1.37, 2.31)	6.25* (4.16, 9.39)	16,865
Ecstasy	1.46* (1.16, 1.85)	5.52* (3.78, 8.06)	16,822
Prescription pain medication	1.53* (1.29, 1.84)	2.75* (1.89, 4.02)	16,846
Ritalin or Adderall	1.40* (1.07, 1.82)	4.96* (3.29, 7.49)	16,779
Cold medicine	1.33* (1.12, 1.58)	1.40 (0.97, 2.02)	16,824
Other drug	1.44* (1.18, 1.75)	3.17* (2.15, 4.66)	16,827
Past 30 days			
Cigarettes	1.66* (1.33, 2.07)	4.78* (3.23, 7.06)	16,798
Tobacco	1.41 (0.90, 2.21)	7.71* (4.77, 12.45)	16,821
Alcohol (1 drink)	1.25* (1.05, 1.48)	2.47* (1.70, 3.59)	16,795
Heavy episodic drinking	1.53* (1.26, 1.85)	3.13* (2.13, 4.61)	16,824
Marijuana	1.56* (1.30, 1.86)	2.92* (2.01, 4.26)	16,815
Inhalants	1.31* (0.95, 1.80)	6.25* (4.06, 9.64)	16,808
Prescription pain medication	1.62* (1.27, 2.06)	5.62* (3.78, 8.36)	16,849
Other drug	1.62* (1.26, 2.09)	4.22* (2.75, 6.48)	16,834
In school			
Cigarettes	1.69* (1.18, 2.42)	6.54* (4.11, 10.42)	16,831
Tobacco	1.81* (1.15, 2.85)	7.68* (4.61, 12.79)	16,812
Alcohol	1.44* (1.13, 1.85)	3.92* (2.60, 5.92)	16,802
Marijuana	1.52* (1.12, 2.06)	5.38* (3.46, 8.36)	16,832
Other illegal drugs	1.03 (0.86, 1.23)	1.26 (0.84, 1.87)	16,757

* *p* < .001.

As seen in Table 3, results from the logistic regression analyses also indicated that both categories of homeless LGB students were significantly more likely to report past‐30‐day and in‐school substance use (all models). Acute homeless LGB students again had the highest risk. For example, acute homeless students were about 3 times as likely to report heavy episodic drinking (5 or more alcoholic drinks in a row; AOR = 3.13; 95% CI = 2.13, 4.61) and more than 5 times as likely to smoke marijuana in school (AOR = 5.38; 95% CI = 3.46, 8.36), compared to housed LGB students. In addition, acute homeless LGB students were more than 6 times as likely to use inhalants in the past 30 days (AOR = 6.25; 95% CI = 4.06, 9.64). Doubled‐up LGB students had an increased likelihood of smoking cigarettes on school grounds (AOR = 1.69; 95% CI = 1.18, 2.42) and almost 50% more likely to drink alcohol on school grounds (AOR = 1.44; 95% CI = 1.13, 1.85).

## DISCUSSION

This study adds to the limited research on the intersection of student homelessness, sexual orientation, and substance use. The goals of this study were to explore differences between housed LGB students and LGB students experiencing homelessness, in substance use on and off school grounds and the relationship between homelessness and substance use in the LGB student population. Considering that this study utilized a representative statewide sample, several alarming findings emerged from the analyses: (a) LGB students experiencing homelessness presented higher rates of all substance use patterns compared with housed LGB students, and (b) a strong association existed between being a homeless student, especially in the category of acute homelessness, and substance use on school grounds.

The findings highlight differences in substance use patterns between LGB students experiencing homelessness and housed LGB students. Lifetime and past‐30‐day use rates were higher for all substances among LGB students experiencing homelessness, both doubled‐up and acute homeless youth, compared to their housed peers. To some extent, this has been supported in previous research that compared heterosexual and LGBT homeless youth.^6^, ^29^ The current investigation provided a nuanced exploration of the topic using a large‐scale representative sample. First, substances in different categories (stimulants, depressants, opioid‐related painkillers, and hallucinogens) were examined, which allows for a better understanding of the challenges faced by these youth and illuminates the need for different types of interventions based on the substance used and the implications of their use among the LGB housed and homeless students. For instance, in examining past‐30‐day use, LGB students who experienced acute homelessness were much more likely to use inhalants and prescription pain medications compared with LGB students who were doubled up or housed. Second, the exploration of both lifetime use and past‐30‐day use allowed for an understanding of the differences between LGB students who experimented several times with a substance and those who may have addiction problems. The findings indicate that LGB students who experienced acute homelessness were at much higher risk of substance use problems, in the context of various types of substances.

Additionally, the findings indicate that LGB homeless and housed students use substances in school. LGB students experiencing homelessness reported much higher rates than LGB housed students of all substances used in school. This finding is alarming because attending school is often considered a protective factor for homeless youth,^30^ yet the analyses paint a more complex picture. Attending school by itself may not serve as a protective factor; on the contrary, it could be a place where substances are offered to students and where substance use takes place. However, the school context also provides a unique opportunity for prevention and intervention. The ESSA and MVA require that districts and schools collect information about student homelessness and allocate funding to support these students.^22^, ^27^ The analyses provided novel information regarding the subpopulation of LGB students experiencing homelessness, which can guide the design of programs and services to support homeless students, particularly LGB students experiencing homelessness.

Moreover, the differences between LGB homeless students who experienced acute homelessness and those who were doubled up should be considered. The findings clearly indicate that those who experienced acute homelessness presented higher rates of substance use compared with those who were doubled up or housed. Yet students who were doubled up still had higher risk of substance use compared with housed LGB students. The doubled up category is often overlooked in the literature, specifically in considering the intersection with sexual orientation.^31^, ^32^ It could be that different supports should be offered to different groups and that prevention, intervention, and rehabilitation efforts should be designed accordingly.

Even though the data were collected prior to the COVID‐19 pandemic, the findings provide valuable insights regarding the LGB homeless student population, which should be considered when designing programs for prevention and intervention for this at‐risk group during this time. Although studies are limited on this topic due to the recent emergency of the pandemic, existing findings suggest there is reason for concern regarding the increase in substance use among adolescents.^33^, ^34^ Additionally, both LGB youth and homeless youth are vulnerable pediatric groups that face unique risks because of the pandemic and its implications.^35^, ^36^ These risks include home isolation, given home can be an unsafe place or nonexistent for those who experience acute homelessness; economic and family stressors; and lack of social support and engagement.^35^, ^36^ These factors are likely to increase the risk of substance use and additional harmful behaviors.

### Limitations

Findings of this study should be understood in the context of several limitations. The design of this study was cross‐sectional and hence, causal relationship between homelessness and substance use among LGB students could not be assessed. Further research is needed to explore this causal relationship, as well as that between being a sexual minority and homelessness. There is still a gap in the research and conflicting findings regarding causality between substance use and homelessness in general, and the same goes for LGB youth.^37^ Moreover, additional nuanced information is needed to better understand and serve this group. In particular, the CHKS data do not include an item differentiated between accompanied and unaccompanied LGB homeless youth, which could point to different needs in each group. Additionally, the survey includes a question about housing only for 9th and 11th grades, although federal data show that most homeless students are in elementary school. Previous research shows that compared to housed students, homeless students start using substances before the age of 10 in a much higher rate.^24^ Future research is needed to further explore these findings, including the intersection with sexual orientation.

## IMPLICATIONS FOR SCHOOL HEALTH POLICY AND FUTURE RESEARCH DIRECTIONS

The study's findings contribute to different research areas and underline the importance of exploring the intersection of homelessness, sexual orientation, school, and risk behaviors. Furthermore, the findings have valuable implications for schools, school districts, policy makers, and service providers who work with LGB youth that experience homelessness. Specifically, the findings highlight that schools are an important context for homeless LGB students, that should be further explored regarding additional risk and protective factors. The findings suggest that schools are locations in which LGB students experiencing homelessness may consume and be offered substances. The present study adopted an epidemiological perspective as a foundational step for better understanding substance use patterns for homeless LGB students, specifically substance use in school. Additional studies are needed that explore the role of school for these students regarding the major challenges they face in terms of social, health, and behavioral risks. Particularly, we need a better understanding of whether schools serve as a protective or risk factor in the lives of LGB students who experience homelessness and what additional factors are associated with their risk of substance use.
